# Predictors of treatment response to liraglutide in type 2 diabetes in a real-world setting

**DOI:** 10.1007/s00592-018-1124-0

**Published:** 2018-03-12

**Authors:** N. Simioni, C. Berra, M. Boemi, A. C. Bossi, R. Candido, G. Di Cianni, S. Frontoni, S. Genovese, P. Ponzani, V. Provenzano, G. T. Russo, L. Sciangula, A. Lapolla, C. Bette, M. C. Rossi, ReaL (NN2211-4118) Study Group*, Natalino Simioni, Cesare Berra, Massimo Boemi, Antonio Carlo Bossi, Riccardo Candido, Graziano Di Cianni, Simona Frontoni, Stefano Genovese, Paola Ponzani, Vincenzo Provenzano, Giuseppina Russo, Luigi Sciangula, Annunziata Lapolla, Cristiano Bette, Maria Chiara Rossi

**Affiliations:** 1 Presidio Ospedaliero di Cittadella, Cittadella, Padua, Italy; 2 Humanitas Research Hospital, Rozzano, MI Italy; 3 IRCCS INRCA, Ancona, Italy; 4 ASST Bergamo Ovest, Treviglio, BG Italy; 5 Azienda Sanitaria Universitaria Integrata di Trieste, Trieste, Italy; 6 Ospedale di Livorno, Livorno, Italy; 7 University of Rome Tor Vergata, Rome, Italy; 8 IRCCS Centro Cardiologico Monzino, Milan, Italy; 9 Ospedale La Colletta, ASL3 Genovese, Arenzano, Italy; 10 Centro Regionale di Riferimento Diabetologia ed Impianto Microinfusori Sicilia, Partinico, Palermo, Italy; 11 University of Messina, Messina, Italy; 12 IRCCS Multimedica - Ospedale di Castellanza, Varese, Italy; 13 University of Padua, Padua, Italy; 14 Novo Nordisk Spa, Rome, Italy; 15 CORESEARCH – Center for Outcomes Research and Clinical Epidemiology, Via Tiziano Vecellio, 2, 65124 Pescara, Italy

**Keywords:** Liraglutide, Type 2 diabetes, Response to therapy, RECPAM analysis, GLP-1RA

## Abstract

**Aims:**

There is an unmet need among healthcare providers to identify subgroups of patients with type 2 diabetes who are most likely to respond to treatment.

**Methods:**

Data were taken from electronic medical records of participants of an observational, retrospective study in Italy. We used logistic regression models to assess the odds of achieving glycated haemoglobin (HbA_1c_) reduction ≥ 1.0% point after 12-month treatment with liraglutide (primary endpoint), according to various patient-related factors. RECursive Partitioning and AMalgamation (RECPAM) analysis was used to identify distinct homogeneous patient subgroups with different odds of achieving the primary endpoint.

**Results:**

Data from 1325 patients were included, of which 577 (43.5%) achieved HbA_1c_ reduction ≥ 1.0% point (10.9 mmol/mol) after 12 months. Logistic regression showed that for each additional 1% HbA_1c_ at baseline, the odds of reaching this endpoint were increased 3.5 times (95% CI: 2.90–4.32). By use of RECPAM analysis, five distinct responder subgroups were identified, with baseline HbA_1c_ and diabetes duration as the two splitting variables. Patients in the most poorly controlled subgroup (RECPAM Class 1, mean baseline HbA_1c_ > 9.1% [76 mmol/mol]) had a 28-fold higher odds of reaching the endpoint versus patients in the best-controlled group (mean baseline HbA_1c_ ≤ 7.5% [58 mmol/mol]). Mean HbA_1c_ reduction from baseline was as large as − 2.2% (24 mol/mol) in the former versus − 0.1% (1.1 mmol/mol) in the latter. Mean weight reduction ranged from 2.5 to 4.3 kg across RECPAM subgroups.

**Conclusions:**

Glycaemic response to liraglutide is largely driven by baseline HbA_1c_ levels and, to a lesser extent, by diabetes duration.

## Introduction

Liraglutide is a once-daily human glucagon-like peptide-1 (GLP-1) analogue available for the treatment of type 2 diabetes (T2D), and its efficacy and safety have been demonstrated in the Liraglutide Effect and Action in Diabetes (LEAD) study programme [1–7]. Liraglutide has also cardioprotective benefits in patients with T2D at increased risk of cardiovascular disease [8]. Liraglutide was approved in the EU in 2009, and data from real-world observational studies have further demonstrated that the benefits of liraglutide on glycated haemoglobin (HbA_1c_) and body weight loss were consistent with those obtained in the randomised LEAD trials [9]. Long-term studies indicated that the benefits were sustained for up to 3 years [10, 11].

Liraglutide has been demonstrated to have benefits across a diverse spectrum of patients with T2D, but the extent of HbA_1c_ improvement differs within patient groups having different demographics and clinical characteristics [12]. Thus, there is an unmet need to identify subgroups of patients with T2D receiving liraglutide who are most likely to have the greatest response to treatment. This information would help healthcare providers individualise treatment options and assess cost benefits. Patients and healthcare professionals could benefit from a more detailed understanding of factors associated with improved response to liraglutide.

The ReaL study (ClinicalTrials.gov identifier: NCT02255266) was the largest observational study of liraglutide in Italian clinical practice, showing that 43.5% of patients achieved HbA_1c_ reduction ≥ 1% (10.9 mmol/mol) after 12 months of treatment (primary endpoint). This manuscript reports findings from a secondary analysis performed to identify subgroups or classes of patients with T2D who were more likely to have an improved response to liraglutide owing to specific combinations of clinical and socio-demographic characteristics.

## Materials and methods

ReaL was an observational, retrospective, longitudinal, multicentre study involving 45 Italian diabetes clinics throughout the country. The design and methods of this real-world study have been previously reported [13]. Briefly, all consecutive patients aged ≥ 18 years diagnosed with T2D and receiving their first prescription of liraglutide in 2011 were eligible for the study. This study was conducted in accordance with the Declaration of Helsinki (last amended by 59th WMA General Assembly, Seoul, October 2013) and the Guidelines for Good Pharmacoepidemiology Practices (ICH-GPP Revision 2, April, 2007). A written informed consent, approved by an independent ethics committee, was signed by all patients before data collection. Data on a range of key clinical variables were obtained from electronic medical records. Information on fasting plasma glucose (FPG), body weight, body mass index (BMI), diabetes duration, presence of diabetes complications, liraglutide treatment, and treatment with other oral antidiabetic drugs (OADs) was extracted at the date of the first liraglutide prescription at baseline in 2011 and after 12 months. The frequency of patients achieving HbA_1c_ reduction ≥ 1% (10.9 mmol/mol) after 12 months’ treatment (primary endpoint) was calculated. This primary endpoint was selected because it represents a mean effect seen in randomised clinical trials of liraglutide and is a strong indicator of effectiveness that is meaningful to both patients and clinicians. It is also in line with the trend in clinical care to individualise specific HbA_1c_ targets. Information on side effects and adverse events was not explored, since it was not available in the electronic medical records in a standardised format.

### Statistical analysis

Results are expressed as mean and standard deviation (SD) for continuous variables, and proportion and percentages for categorical measures, respectively. Between-group patient characteristics were compared with a Mann–Whitney *U* test or Student’s *t* test (as appropriate) for continuous variables, or a Chi-square test for categorical variables. Univariate logistic regression was used to identify baseline characteristics of patients who achieved the primary endpoint (HbA_1c_ reduction ≥ 1.0% [10.9 mmol/mol] at 12 months), compared with those who did not.

Multivariate logistic regression analysis was performed to identify independent factors associated with the endpoint after adjustment for other variables. Covariates included in the multivariate analysis were age, sex, diabetes duration, baseline HbA_1c_, FPG, BMI, presence of diabetes complications, treatment at the first prescription of liraglutide (baseline), treatment modality, liraglutide dose, hypertension, dyslipidaemia, and estimated glomerular filtration rate (eGFR) levels. Standardised criteria which were used for diagnosis of hypertension were not established a priori for this study. Data were collected from electronic medical records, but in the Italian national guidelines, hypertension and dyslipidaemia cut-offs are blood pressure (BP) values ≥ 140/90 mmHg and low-density lipoprotein (LDL)-cholesterol ≥ 100 mg/dl, respectively. Covariates used in the multivariate analysis were chosen based on clinical judgment and did not depend on reaching statistical significance in the univariate analysis. Results are shown as odds ratios (ORs) and 95% confidence intervals (CI).

RECursive Partitioning and AMalgamation (RECPAM) analysis, a tree-based statistical method that integrates standard regression and tree-growing techniques, was used to detect potential interactions among the different variables in predicting reduction of at least 1% in HbA_1c_ and identify homogeneous and distinct subgroups of patients with increased likelihood of reaching the endpoint [14]. In diabetes, RECPAM analysis has been previously used to identify: patients with T2D at risk of microalbuminuria [15], factors associated with impaired quality of life in patients using continuous subcutaneous insulin infusion [16], and patients at higher risk of cardiovascular disease [17]. The RECPAM analysis was performed using SAS^®^ (Release 9.4 Cary, NC, USA) and a macro-routine written by F. Pellegrini and updated by M. Scardapane and G. Lucisano. At each partitioning step, the RECPAM method automatically chose the covariate and best binary split to maximise the difference in risk of experiencing the outcome. The algorithm stopped when user-defined stopping rules were met. In this case, each final class was required to have at least 100 patients in total and 30 patients with the target endpoint.

The set of variables tested in the RECPAM analysis was the same tested in the multivariate logistic regression analysis. Continuous variables were not categorised so as to allow the algorithm to choose the natural cut-off points when identifying distinct subgroups of patients. For each subgroup or class, the proportions (%) of patients reaching the endpoint and the likelihood (ORs and 95% CI) to reach the endpoint versus the reference subgroup were obtained. Finally, to detect additional global correlates (i.e. variables playing a role for all patients, irrespective of the interactions detected by RECPAM), a logistic regression model with RECPAM-identified subgroups and all the covariates ruled out by the algorithm was performed. No imputation was used for missing data, and sensitivity analyses were not performed.

## Results

A total of 1723 patients were included in the analysis. Baseline characteristics, including diabetes complications and prior treatment regimens, are shown in Table 1. At baseline, most patients were being treated with metformin, either as monotherapy (*n* = 803, 46.6%) or with sulphonylureas (*n* = 457, 26.5%). Few patients (*n* = 100, 5.8%) received insulin. Most patients received liraglutide as an add-on to previous therapies (63.2%), with 33.4% replacing another prior drug with liraglutide, and 3.4% reducing the number of prior therapies. Mean BMI at baseline was 35.6 ± 5.9 kg/m^2^, with 83.3% of patients considered to have obesity (BMI > 30 kg/m^2^).

**Table 1:** Baseline characteristics of 1723 patients with type 2 diabetes prior to starting liraglutide treatment

Variable	Category	Value
Age (years)		58.9 ± 9.5
Sex (%)	Female	45.1
Diabetes duration (years)		9.6 ± 7.1
HbA_1c_ (% points)		8.3 ± 1.4 (67 ± 15.3 mmol/mol)
Fasting plasma glucose (mg/dL)		171.8 ± 52.2
BMI (kg/m^2^)		35.6 ± 5.9
Presence of diabetes complications (%)		
Coronary heart disease	No	86.9
	Yes	13.1
Stroke	No	98.1
	Yes	1.9
Peripheral vascular disease	No	93.3
	Yes	6.7
Diabetic retinopathy	No	81.5
	Yes	18.5
Sensory-motor neuropathy	No	86.5
	Yes	13.5
Baseline treatment (%)	Metformin	46.6
	Other monotherapy	7.6
	Metformin + SU	26.5
	Other dual	8.6
	≥3 OADs	3.7
	Insulin ± OADs	7
Liraglutide treatment modality (%)	Switch	33.4
	Add-on	63.2
	Reduce	3.4
Systolic blood pressure (mmHg)		139.3 ± 18.1
Diastolic blood pressure (mmHg)		81.3 ± 10.0
Hypertension (≥ 140/90 mmHg) (%)	No	39.8
	Yes	60.2
Total cholesterol (mg/dL)		180.8 ± 39.8
HDL-cholesterol (mg/dL)		45.0 ± 10.9
LDL-cholesterol (mg/dL)		102.9 ± 35.3
Dyslipidaemia (%)	No	34.4
	Yes	65.6
eGFR (%)	≤ 30 mL/min/1.73 m^2^	0.1
	> 30– < 60 mL/min/1.73 m^2^	11.4
	≥ 60– < 90 mL/min/1.73 m^2^	43.1
	≥ 90 mL/min/1.73 m^2^	45.4

Values are mean ± SD or %

Add-on, liraglutide added to prior therapy; BMI, body mass Index; eGFR, estimated glomerular filtration rate (using the Chronic Kidney Disease-Epidemiology Collaboration formula); HbA_1c_, glycated haemoglobin; HDL, high-density lipoprotein; LDL, low-density lipoprotein; reduce, number of prior OADs was reduced with addition of liraglutide; OAD, oral antidiabetic drug; SU, sulphonylurea; switch, switch to liraglutide from prior therapy

By 12 months (primary endpoint analysis), a total of 194/1723 (11.2%) patients had discontinued liraglutide treatment. For those with a known reason (*n* = 166), most (*n* = 75/166) were owing to lack of effectiveness. An additional 35 discontinued due to liraglutide intolerance, 28 owing to gastrointestinal side effects, and 20 discontinued for other reasons. A total of 19 patients were non-adherent to therapy. At 12 months, there were 1325 (76.9%) patients with HbA_1c_ values available at both baseline and 12 months, and 577/1325 (43.5%) reached the primary endpoint (HbA_1c_ reduction ≥ 1.0% [10.9 mmol/mol]).

Patients who reached the endpoint had a shorter mean diabetes duration (9.1 ± 6.9 vs. 10.0 ± 7.0 years, *p *= 0.04), higher mean HbA_1c_ at baseline (9.0 ± 1.4 [75 ± 15.3 mmol/mol] vs. 7.7 ± 1.0% [61 ± 10.9 mmol/mol], *p *< 0.0001), higher mean diastolic BP (82.6 ± 10.0 vs. 80.3 ± 9.8 mmHg, *p *= 0.0002) and higher mean total cholesterol levels (183.1 ± 41.8 vs. 177.2 ± 37.4 mg/dL, *p *= 0.02) compared to those who failed to reach the primary endpoint. Mean BMI was nearly identical in the two groups (35.6 ± 5.8 vs. 35.5 ± 5.8 kg/m^2^, *p *= 0.72), and there were no significant differences in mean high-density lipoprotein (HDL)-cholesterol (*p *= 0.11) or mean LDL-cholesterol (*p *= 0.16). There were no significant differences between the two groups in the proportion of patients using antihypertensive or lipid-lowering medications or other diabetes treatments at baseline.

### Logistic regression analysis

The odds of achieving the primary endpoint, by patient characteristic, are shown in Table 2. In the univariate analysis, higher HbA_1c_ at baseline was associated with significantly higher odds (OR 2.78; 95% CI [2.43; 3.18]; *p *< 0.0001). Shorter diabetes duration was associated with a significantly lower odds of reaching the endpoint (OR 0.98; 95% CI [0.97; 1.00]; *p *= 0.04). Higher diastolic BP (OR 1.02; 95% CI [1.01; 1.04]; *p *= 0.0002) and higher total cholesterol (OR 1.00; 95% CI [1.00; 1.01]; *p =* 0.0203) were also associated with significantly increased odds of reaching the endpoint. Other patient characteristics, such as age, sex, BMI, presence of various diabetes complications, dyslipidaemia or eGFR levels, were not significantly associated with odds of reaching the endpoint.

**Table 2:** Univariate and multivariate analysis of factors predicting reduction of HbA_1c_ ≥ 1.0% (10.9 mmol/mol) among 1325 patients^a^ after 12 months of treatment with liraglutide

Variable	Category	Univariate logistic regression		Multivariate logistic regression	
		Odds ratio (95% CI)	*p*-value	Odds ratio (95% CI)^b^	*p*-value
Age	N/A	1.00 (0.99; 1.01)	0.9689	1.02 (1.00; 1.04)	0.02
Diabetes duration (years, continuous)	N/A	0.98 (0.97; 1.00)	0.04	0.97 (0.94; 0.99)	0.007
HbA_1c_ (continuous)	N/A	2.78 (2.43; 3.18)	< 0.0001	3.52 (2.90; 4.27)	< 0.0001
BMI kg/m^2^ (continuous)	N/A	1.00 (0.98; 1.02)	0.7207	1.01 (0.98; 1.03)	0.61
Baseline treatment	Metformin	1.00^c^		1.00^c^	N/A
	Other monotherapy	1.17 (0.76; 1.80)	0.4651	0.91 (0.52; 1.59)	0.75
	Metformin + SU	1.01 (0.77; 1.32)	0.9528	0.50 (0.34; 0.72)	0.0002
	Other dual	1.01 (0.67; 1.52)	0.9615	0.59 (0.34; 1.02)	0.06
	≥ 3 OADs	1.12 (0.62; 2.02)	0.7025	0.41 (0.19; 0.88)	0.02
	Insulin ± OADs	1.00 (0.63; 1.58)	0.9963	0.44 (0.23; 0.85)	0.02
Liraglutide dose	1.8	1.00^c^		1.00^c^	N/A
	1.2	1.43 (1.12; 1.82)	0.0037	1.91 (1.40; 2.61)	< 0.0001
Liraglutide treatment modality	Switch	1.00^c^		1.00^c^	N/A
	Add-on	1.74 (1.38; 2.20)	< 0.0001	1.86 (1.38; 2.51)	< 0.0001
	Reduce	0.56 (0.26; 1.21)	0.1418	0.62 (0.24; 1.59)	0.32
Sex	Female	1.00^c^			
	Male	1.09 (0.88; 1.35)	0.4459		
Fasting plasma glucose (mg/dL, continuous)	N/A	1.01 (1.01; 1.02)	< 0.0001		
Diabetic retinopathy	No	1.00^c^			
	Yes	1.17 (0.87; 1.57)	0.2896		
Sensory-motor neuropathy	No	1.00^c^			
	Yes	1.10 (0.79; 1.52)	0.5731		
Coronary heart disease	No	1.00^c^			
	Yes	0.85 (0.62; 1.18)	0.3408		
Stroke	No	1.00^c^			
	Yes	0.82 (0.38; 1.76)	0.6052		
Peripheral vascular disease	No	1.00^c^			
	Yes	0.85 (0.55; 1.32)	0.4702		
Blood pressure (mm Hg)	≤ 130/80	1.00^c^			
	131–139/81–89	1.25 (0.77; 2.03)	0.3652		
	≥ 140/90	1.11 (0.86; 1.44)	0.4247		
Systolic BP (mm Hg, continuous)	N/A	1.00 (1.00; 1.01)	0.5617		
Diastolic BP (mm Hg, continuous)	N/A	1.02 (1.01; 1.04)	0.0002		
Hypertension	No	1.00^c^			
	Yes	0.91 (0.69; 1.19)	0.4815		
Total cholesterol (mg/dL, continuous)	N/A	1.00 (1.00; 1.01)	0.0203		
HDL-cholesterol (mg/dL, continuous)	N/A	1.0 (1.0; 1.0)	0.1091		
LDL-cholesterol (mg/dL, continuous)	N/A	1.0 (1.0; 1.0)	0.1566		
Dyslipidaemia	No	1.00^c^			
	Yes	0.98 (0.77; 1.24)	0.8573		
eGFR	> 90	1.00^c^			
	61–90	0.97 (0.73; 1.29)	0.8471		
	31–60	0.63 (0.39; 1.02)	0.0603		
	0–30	nc	nc		

Add-on, liraglutide added to prior therapy; BMI, body mass index; BP, blood pressure; CI, confidence interval; eGFR, estimated glomerular filtration rate; HbA_1c_, glycated haemoglobin; LDL, low-density lipoprotein; N/A, not applicable; nc, not calculated; OAD, oral antidiabetic drug; reduce, number of prior OADs was reduced with addition of liraglutide; SU, sulphonylurea; switch, switch to liraglutide from prior therapy

^a^Patients who had HbA_1c_ data recorded at 12 months

^b^Adjusted for age, sex, duration of diabetes, baseline HbA_1c_, FPG, BMI, presence of diabetes complications, hypertension, dyslipidaemia, eGFR levels, treatment scheme at the first prescription of liraglutide, treatment modality, and liraglutide dosage

^c^Reference category

Prior treatment (including insulin) was not significantly associated with reaching the primary endpoint (*p *> 0.05). However, after adjusting for potential confounding in the multivariate analysis, all prior treatment regimens (except for other dual therapy, *p *= 0.06) were associated with a significantly lower odds of achieving the endpoint compared with metformin monotherapy (Table 2). Regarding treatment modality, patients who had liraglutide added to their prior therapy had a significantly higher odds of achieving the primary endpoint (OR 1.74 95% CI [1.38; 2.20]; *p *< 0.0001) compared with patients who switched to liraglutide from their previous therapy. Those results were confirmed in the multivariate analysis.

The proportion of patients using liraglutide at higher doses increased with successive follow-up, with over a third (36.1%) using 1.8 mg at 12 months compared to 5.3% at baseline. Patients using liraglutide 1.2 mg had an increased odds (OR 1.43; 95% CI [1.12; 1.82]; *p *= 0.0037) of reaching the endpoint compared to those using the highest dose (1.8 mg).

### RECPAM analysis

**Fig. 1:**
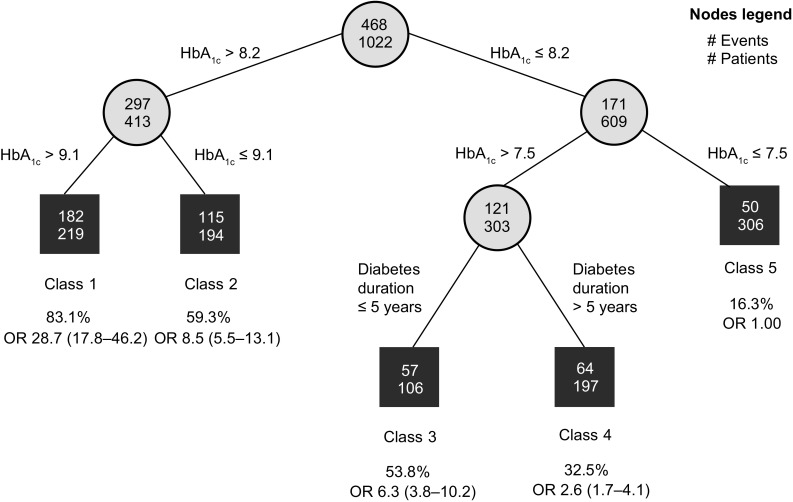
Subgroups of patients with type 2 diabetes with different odds of achieving a HbA_1c_ reduction ≥ 1.0% (10.9 mmo/mol) after 12 months of treatment with liraglutide, identified using RECPAM analysis. The tree-growing algorithm modelled the odds for achieving HbA_1c_ reduction ≥ 1.0%-point using multivariate logistic regression. Splitting variables were automatically selected by the RECPAM routine among the covariates used in the multivariate analysis and are shown between branches. Cut-offs sending patients to the left or right sibling were also automatically chosen by the RECPAM routine and are reported on the relative branches.  %, proportion of patients in subgroup achieving a reduction in HbA_1c_ ≥ 1.0% (10.9 mmol/mol); circles indicate subgroups of patients and squares indicate final RECPAM classes. Numbers inside circles and squares indicate number of patients achieving HbA_1c_ reduction ≥ 1.0% (10.9 mmol/mol). HbA_1c_, glycated haemoglobin; OR, unadjusted odds ratio (95% confidence interval); RECPAM, RECursive Partitioning and AMalgamation

The RECPAM analysis identified five distinct patient subgroups or classes with increasing odds of achieving an HbA_1c_ reduction ≥ 1.0% (10.9 mmol/mol) after 12 months (Fig. 1, Table 3). The proportion of patients reaching the endpoint ranged from 16.3% (reference group) to 83.1%. The splitting variables indicated that baseline HbA_1c_ and, to some extent, diabetes duration were the primary drivers of degree of response to liraglutide, whereas other patient-related factors were not identified as important in discriminating responder subgroups. With patients having baseline HbA_1c_ ≤ 7.5% (58 mmol/mol) considered the reference class (OR = 1.00), the odds of patients in the other classes achieving the endpoint were: Class 4: OR 2.6; 95% CI [1.7; 4.1], patients with HbA_1c_ between 7.5% (58 mmol/mol) and 8.2% (66 mmol/mol), diabetes duration > 5 years; Class 3: OR 6.3; 95% CI [3.8; 10.2], HbA_1c_ between 7.5% (58 mmol/mol) and 8.2% (66 mmol/mol), diabetes duration < 5 years; Class 2: OR 8.5; 95% CI [5.5; 13.1], HbA_1c_ between 8.2% (66 mmol/mol) and 9.1% (76 mmol/mol); and Class 1: OR 28.7; 95% CI [17.8; 46.2], HbA_1c_ > 9.1%.

**Table 3:** Clinical characteristics, at baseline and after 12 months of treatment with liraglutide, by RECPAM class

	RECPAM classification					
	Class 1 *n* = 219	Class 2 *n* = 194	Class 3 *n* = 106	Class 4 *n *= 197	Class 5 *n* = 306	*p*-value
Splitting variables	HbA_1c_ > 9.1% [76 mmol/mol]	8.2% < HbA_1c_ ≥ 9.1% [66 < HbA_1c_ ≥ 76 mmol/mol]	7.5% < HbA1_c_ ≥ 8.2% [58 < HbA_1c_ ≥ 66 mmol/mol] Diabetes duration ≤ 5 years	7.5% < HbA_1c_ ≥ 8.2% [58 < HbA_1c_ ≥ 66 mmol/mol] Diabetes duration > 5 years	HbA_1c_ ≤ 7.5% [58 mmol/mol]	
Unadjusted odds of HbA_1c_ being reduced by ≥ 1.0%	28.7 (17.8; 46.2)	8.5 (5.5; 13.1)	6.3 (3.8; 10.2)	2.6 (1.7; 4.1)	1.00^a^	
Patient characteristic						
Baseline HbA_1c_ (%)	10.2 ± 1.0 [88 ± 10.9 mmol/mol]	8.7 ± 0.3 [72 ± 3.3 mmol/mol]	7.9 ± 0.2 [63 ± 2.2 mmol/mol]	7.9 ± 0.2 [63 ± 2.2 mmol/mol]	7.0 ± 0.5 [53 ± 5.5 mmol/mol]	< 0.0001
Change in HbA_1c_ (%)	− 2.2 ± 1.5 [88 ± 16.4 mmol/mol]	− 1.0 ± 1.1 [88 ± 12.0 mmol/mol]	− 0.9 ± 1.0 [88 ± 10.9 mmol/mol]	− 0.5 ± 0.9 [88 ± 9.8 mmol/mol]	− 0.1 ± 0.8 [88 ± 8.7 mmol/mol]	< 0.0001
Baseline FPG (mg/dl)	223.0 ± 56.7	181.5 ± 41.1	157.3 ± 28.9	159.7 ± 33.2	137.5 ± 28.5	< 0.0001
Change in FPG (mg/dl)	− 59.1 ± 63.7	− 28.9 ± 49.9	− 20.6 ± 40.3	− 14.4 ± 35.5	− 7.1 ± 33.0	0.0002
Baseline BMI (Kg/m^2^)	35.6 ± 5.6	35.3 ± 5.6	37.2 ± 6.3	34.1 ± 5.6	35.7 ± 6.2	< 0.0001
Change in BMI (Kg/m^2^)	− 0.9 ± 2.2	− 1.6 ± 2.0	− 1.3 ± 1.9	− 1.1 ± 1.7	− 1.3 ± 2.1	0.02
Baseline weight (Kg)	101.5 ± 18.5	98.3 ± 17.7	103.9 ± 19.1	93.9 ± 17.4	100.2 ± 19.2	< 0.0001
Change in weight (Kg)	− 2.5 ± 6.1	− 4.3 ± 5.3	− 3.7 ± 5.2	− 3.1 ± 4.7	− 3.7 ± 5.8	0.03
Age (years)	57.7 ± 9.4	60.7 ± 8.0	56.0 ± 9.1	61.2 ± 9.3	59.2 ± 8.9	< 0.0001
Sex (% male)	57.5	55.2	48.1	52.3	56.9	0.46
Duration diabetes (years)	10.2 ± 6.9	11.2 ± 7.3	2.9 ± 1.5	12.1 ± 6.3	9.1 ± 6.8	< 0.0001
Baseline treatment (%)						< 0.0001
Metformin only	34.7	34	71.7	38.6	60.8	
Other monotherapy	7.3	7.7	8.5	8.6	7.2	
Metformin + SU	35.6	35.6	13.2	31.5	14.4	
Other dual therapies	7.3	11.3	3.8	7.6	10.1	
≥ 3 OADs	5.5	4.1	1.9	5.6	2.6	
Insulin ± OADs	9.6	7.2	0.9	8.1	4.9	
Treatment modality (%)						0.34
Switch	31.5	37.6	34.0	36.5	38.2	
Add-on	67.1	59.8	65.1	61.4	57.8	
Reduction	1.4	2.6	0.9	2.0	3.9	
Liraglutide dosage (%)						0.0007
0.6	4.1	4.6	5.7	5.1	7.8	
1.2	55.3	50.5	65.1	58.9	66.7	
1.8	40.6	44.8	29.2	36.0	25.5	
Baseline SBP (mmHg)	142.0 ± 18.4	140.3 ± 16.6	138.0 ± 17.9	140.3 ± 18.7	137.4 ± 16.8	0.09
Change in SBP (mmHg)	− 4.2 ± 18.5	− 2.6 ± 16.7	− 4.4 ± 16.0	− 6.3 ± 19.2	− 5.4 ± 17.6	0.57
Baseline DBP (mmHg)	83.5 ± 10.6	81.2 ± 9.4	81.7 ± 10.1	81.0 ± 9.6	80.0 ± 10.0	0.02
Change in DBP (mmHg)	− 1.8 ± 11.2	− 0.6 ± 9.6	− 1.0 ± 10.6	− 2.4 ± 11.0	− 1.7 ± 11.1	0.60
Baseline total cholesterol (mg/dl)	187.9 ± 43.6	181.5 ± 36.3	185.2 ± 38.1	175.2 ± 34.9	174.8 ± 38.0	0.007
Change in total cholesterol (mg/dl)	− 16.2 ± 40.1	− 9.8 ± 32.3	− 19.9 ± 39.6	− 7.2 ± 34.7	− 7.1 ± 31.0	0.06
Baseline HDL-cholesterol (mg/dl)	42.9 ± 9.5	45.2 ± 11.5	43.5 ± 10.9	46.5 ± 12.0	44.7 ± 10.4	0.07
Change in HDL-cholesterol (mg/dl)	0.6 ± 7.1	1.6 ± 8.3	1.6 ± 7.5	1.8 ± 8.2	0.9 ± 7.9	0.42
Baseline LDL-cholesterol (mg/dl)	104.2 ± 38.8	104.4 ± 30.7	108.5 ± 36.2	96.9 ± 32.1	101.1 ± 32.8	0.13
Change in LDL-cholesterol (mg/dl)	− 9.4 ± 35.7	− 10.8 ± 30.9	− 20.4 ± 36.2	− 7.3 ± 31.8	− 8.7 ± 30.6	0.15
Baseline triglycerides (mg/dl)	211.6 ± 120.0	169.6 ± 80.1	182.9 ± 81.8	163.7 ± 77.9	150.8 ± 75.6	<0.0001
Change in triglycerides	− 35.4 ± 110.2	− 7.3 ± 85.8	− 16.6 ± 82.2	− 11.4 ± 64.6	− 0.4 ± 60.8	0.002
Baseline albuminuria (mg/l)	73.7 ± 150.3	39.0 ± 92.0	37.2 ± 55.7	40.6 ± 64.9	38.0 ± 84.1	0.07
Change in albuminuria (mg/l)	− 20.0 ± 119.2	0.6 ± 58.6	− 1.2 ± 43.3	− 15.2 ± 75.6	− 13.2 ± 89.7	0.91
Baseline eGFR (%)						0.16
0–60	5.5	8.2	2.8	7.6	8.2	
61–90	62.1	57.2	68.9	64.5	55.2	
> 90	32.4	34.5	28.3	27.9	36.6	

Values are mean ± SD unless otherwise stated

BMI, body mass index; DBP, diastolic blood pressure; FPG, fasting plasma glucose; HbA_1c_, glycated haemoglobin; HDL, high-density lipoprotein; LDL, low-density lipoprotein; n, number of subjects in class; OAD, oral antidiabetic drug; RECPAM, RECursive Partitioning and AMalgamation; SBP, systolic blood pressure; SU, sulphonylurea

^a^Reference category for odds ratio

Although all RECPAM classes showed HbA_1c_ reduction, the patient subgroup with the greatest odds of achieving an HbA_1c_ reduction ≥ 1.0% (10.9 mmol/mol) can be described as having the following: mean HbA_1c_ of 10.2% (88 mmol/mol), mean FPG of 223.0 mg/dL, mean diabetes duration of 10.2 years at baseline, metformin treatment ± sulphonylureas at initiation of liraglutide treatment, and liraglutide as an adjunct to prior therapy (versus discontinuation of prior treatment) (Table 3). Each RECPAM class showed a reduction in mean weight, ranging from 2.5 to 4.3 kg, after 12 months’ treatment with liraglutide. There was no obvious relationship between mean HbA_1c_ reduction and mean weight loss. A final logistic model adjusted with other covariates deemed clinically important and with RECPAM classes forced into the model is shown in Table 4. The final logistic model with both the RECPAM classes and the covariates not entering the tree forced in the model (Table 4) showed that additional global variables associated with the likelihood of reaching the endpoint were baseline treatment scheme, liraglutide dosage and treatment modality.

**Table 4:** Final logistic model^a^ showing key factors predicting reduction of HbA_1c_ ≥ 1.0% [10.9 mmol/mol] among 1325 patients after 12 months of treatment with liraglutide, with RECPAM classes forced in the model

Factor	OR (95% CI)	*p*-value
RECPAM classes		
Class 1	33.69 (18.10–62.74)	< 0.0001
Class 2	10.33 (6.23–17.12)	< 0.0001
Class 3	5.72 (3.35–9.76)	< 0.0001
Class 2	2.89 (1.80–4.65)	< 0.0001
Class 5	1.00^b^	
Baseline treatment		
Other monotherapies	0.93 (0.51–1.69)	0.81
Metformin + sulphonylurea	0.47 (0.31–0.70)	0.0002
Other dual therapies (metformin + TZD, metformin + glinides, SU + TZD)	0.73 (0.40–1.31)	0.29
≥ 3 OADs	0.39 (0.17–0.88)	0.02
Insulin ± OADs	0.47 (0.24–0.94)	0.03
Metformin only	1.00^b^	
Liraglutide dosage (mg)		
0.6	1.02 (0.49–2.12)	0.95
1.2	2.05 (1.45–2.90)	< 0.0001
1.8	1.00^b^	
Liraglutide treatment modality		
Add-on to existing treatment	1.79 (1.29–2.50)	0.0005
Reduction of no. of drug classes	0.52 (0.17–1.63)	0.26
Switch from another drug class	1.00^b^	

BMI, body mass index; CI, confidence interval; eGFR, estimated glomerular filtration rate; FPG, fasting plasma glucose; HbA_1c_, glycated haemoglobin; OAD, oral antidiabetic drug; OR, odds ratio; SU, sulphonylurea; TZD, thiazolidinedione

^a^Model was adjusted for age, sex, FPG, BMI, presence of diabetes complications, hypertension, dyslipidaemia, and eGFR levels

^b^Reference category

## Discussion

This is the first RECPAM analysis to identify distinct groups of patients with T2D who were prescribed liraglutide in routine clinical practice according to their predicted degree of response to liraglutide treatment. These data can improve clinical practice by providing a deeper knowledge of factors influencing liraglutide’s impact on metabolic control. The key message of this analysis is that only baseline HbA_1c_ and to a lesser extent diabetes duration were predictive of liraglutide effectiveness. Furthermore, these results for the first time clarify that HbA_1c_ reduction can exceed 2.0% when baseline levels are > 9.0%. This finding has important clinical and health policy implications for the Italian Drugs Agency (AIFA) regulations, considering that patients with HbA_1c_ ≥ 8.5% are currently excluded from the GLP-1 receptor agonists’ reimbursement policy, which requires HbA_1c_ between 7.5 (58 mmol/mol) and 8.5% (69 mmol/mol) (AIFA regulations).

Different patterns have been reported in clinical trials with regard to dose response with liraglutide. In this study, patients using the 1.2-mg liraglutide dose as maintenance dose were more likely to reach the primary endpoint than those using the higher maintenance dose (1.8 mg). This is likely due to an indication bias because patients struggling to achieve good glycaemic control were up-titrated to the higher dose, but owing to their disease severity, they still did not respond as well as healthier patients who did not require an increased dose. Escalation from the starting liraglutide dose of 0.6–1.2 mg likely occurred earlier after initiation, whereas when escalation to 1.8 mg occurred, it tended to be later in the study.

In line with existing findings [18–20], we found that the higher the baseline HbA_1c_ level, the higher the reduction achieved. Multivariate analysis showed that the likelihood of reaching the endpoint increased by 3.5 times for every 1% HbA_1c_ increase at baseline. In addition, by applying the RECPAM analysis, the study showed that the likelihood of reaching the endpoint was 28 times higher with baseline HbA_1c_ > 9.1% as compared to baseline levels < 7.5%. In the EVIDENCE study [21], conducted in France by general practitioners and specialists, on 2029 patients, there was a mean (± SD) HbA_1c_ reduction from baseline of 1.01 ± 1.54% (from 8.46 ± 1.46 to 7.44% ± 1.20; *p *< 0.0001); after 2 years, 29.9% (95% CI 27.7; 31.2) of patients still had HbA_1c_ ≤ 7.0%; in the cohort treated within specialist care settings (*N* = 1398), HbA_1c_ reduction was − 0.8%.

In the current study, although there were differences in the degree of liraglutide response, each RECPAM class showed decreases in HbA_1c_ from baseline after 12 months of treatment. As might be expected, a greater proportion of patients with the poorest glycaemic control at baseline achieved the primary endpoint of HbA_1c_ reduction ≥ 1.0% (10.9 mmol/mol) after 12 months, since it would be incrementally more difficult to achieve that degree of absolute HbA_1c_ reduction in patients already at or near glycaemic targets. Nevertheless, these results suggest that there is a distinct subgroup of patients for whom liraglutide treatment can help achieve HbA_1c_ reductions in excess of 2.0% (21.9 mmol/mol), a finding that may have important clinical implications.

The RECPAM algorithm selected only baseline HbA_1c_ and diabetes duration as important splitting variables when creating the responder subgroups or classes. This indicated that other patient variables were less important in determining the degree of response to liraglutide. Although BMI was not selected by the algorithm, this too may be because of the high prevalence of obesity in the sample.

Multivariate logistic regression with RECPAM categories forced into the model further confirmed that liraglutide is best used as an add-on to, rather than replacement for, prior treatment regimens (generally OADs) in T2D (OR 1.79; 95% CI [1.29; 2.50]). This finding is in line with current treatment guidelines [22]. Interestingly, the largest patient subgroup (*n* = 306, RECPAM Class 5) (Table 3) had comparatively good HbA_1c_ control (≤ 7.5% [58 mmol/mol]), suggesting that there is also a patient subgroup who may initiate liraglutide to pair the glycaemic control to weight loss.

Regarding the role of diabetes duration, a previous study on liraglutide reported a higher efficacy in patients with short diabetes duration [12], while the ReaL study [13] found improvements in metabolic control also in patients with long diabetes duration. RECPAM analysis clarifies that diabetes duration can play a role mainly for patients with HbA_1c_ levels between 7.5 and 8.2%; in particular, one in two patients with diabetes duration ≤ 5 years reached the endpoint, compared to one in three for a diabetes duration > 5 years. The role of BMI and previous therapy as independent predictors emerging in other studies [19, 23] was not confirmed in our study.

A strength of this study was the large sample size. Use of real-world data also makes the findings more generalisable to patient populations seen in regular clinical practice. The observational nature of the study may introduce bias in the selection of patients who were prescribed liraglutide; however, consecutive enrolment of all patients was adopted to minimise this. Since these results reflect the clinical usage of liraglutide in Italy, they may not be generalisable to countries with different usage patterns. As a retrospective study based on electronic medical records, the completeness of information depended on the ability of participating centres to record clinical data. It should be noted that data completeness was judged satisfactory (i.e. 97.2–56.3% complete for the adjustment variables used). Insulin secretion capacity was not evaluated as a potential predictor of HbA_1c_ reduction with liraglutide, although several studies have suggested the usefulness of this parameter in predicting the effectiveness of liraglutide [24, 25]. This would be useful to explore in future studies. We cannot exclude the involvement of other factors, besides HbA_1c_ and partly diabetes duration, in determining HbA_1c_ reduction through liraglutide, but we analysed all factors easily available to diabetologists to guide routine clinical practice.

In conclusion, in this study, glycaemic response to liraglutide was largely driven by baseline HbA_1c_ levels and to a lesser extent by diabetes duration. The clinical benefit seems to be maximised when used as an add-on to prior therapies. All RECPAM classes showed weight loss, which appeared independent of mean HbA_1c_ reduction. RECPAM analyses suggest an urgent need to revise the AIFA criteria for reimbursement due to the finding that HbA_1c_ reduction can exceed 2.0% in people with HbA_1c_ > 9.0%.

## References

[1] Victoza^®^ summary of product characteristics (2016)

[2] Garber A, Henry R, Ratner R (2009). Liraglutide versus glimepiride monotherapy for type 2 diabetes (LEAD-3 Mono): a randomised, 52-week, phase III, double-blind, parallel-treatment trial. Lancet.

[3] Marre M, Shaw J, Brändle M (2009). Liraglutide, a once-daily human GLP-1 analogue, added to a sulphonylurea over 26 weeks produces greater improvements in glycaemic and weight control compared with adding rosiglitazone or placebo in subjects with Type 2 diabetes (LEAD-1 SU). Diabet Med.

[4] Nauck M, Frid A, Hermansen K (2009). Efficacy and safety comparison of liraglutide, glimepiride, and placebo, all in combination with metformin, in type 2 diabetes: the LEAD (liraglutide effect and action in diabetes)-2 study. Diabetes Care.

[5] Zinman B, Gerich J, Buse JB (2009). Efficacy and safety of the human GLP-1 analog liraglutide in combination with metformin and TZD in patients with type 2 diabetes mellitus (LEAD-4 Met + TZD). Diabetes Care.

[6] Russell-Jones D, Vaag A, Schmitz O (2009). Liraglutide vs insulin glargine and placebo in combination with metformin and sulfonylurea therapy in type 2 diabetes mellitus (LEAD-5 met + SU): a randomised controlled trial. Diabetologia.

[7] Buse J, Rosenstock J, Sesti G (2009). Liraglutide once a day versus exenatide twice a day for type 2 diabetes: a 26-week randomized, parallel-group, multinational, open-label trial (LEAD-6). Lancet.

[8] Marso SP, Daniels GH, Brown-Frandsen K (2016). Liraglutide and cardiovascular outcomes in type 2 diabetes. N Engl J Med.

[9] Ostawal A, Mocevic E, Kragh N, Xu W (2016). Clinical effectiveness of liraglutide in type 2 diabetes treatment in the real-world setting: a systematic literature review. Diabetes Ther.

[10] Ponzani P, Scardapane M, Nicolucci A, Rossi MC (2016). Effectiveness and safety of liraglutide after three years of treatment. Minerva Endocrinol.

[11] Rondinelli M, Rossi A, Gandolfi A (2017). Use of liraglutide in the real world and impact at 36 months on metabolic control, weight, lipid profile, blood pressure, heart rate, and renal function. Clin Ther.

[12] Ratner R, Brett J, Khutoryansky N, Aroda VR (2012). Identifying predictors of response to liraglutide in type 2 diabetes using recursive partitioning analysis. Diabetologia.

[13] Lapolla A, Berra C, Boemi M (2017). Long-term effectiveness of liraglutide for treatment of type 2 diabetes in a real-life setting: a 24-month, multicenter, non-interventional, retrospective study. Adv Ther.

[14] Ciampi A (1992) Constructing prediction trees from data: the RECPAM approach. In: Proceedings from the Prague 1991 summer school on computational aspects of model choice. Physica-Verlag, Heidelberg, pp 105–152

[15] Rossi MC, Nicolucci A, Pellegrini F (2008). Identifying patients with type 2 diabetes at high risk of microalbuminuria: results of the DEMAND (Developing Education on Microalbuminuria for Awareness of reNal and cardiovascular risk in Diabetes) Study. Nephrol Dial Transplant.

[16] Franciosi M, Maione A, Pomili B (2010). Correlates of quality of life in adults with type 1 diabetes treated with continuous subcutaneous insulin injection. Nutr Metab Cardiovasc Dis.

[17] Marzona I, Avanzini F, Lucisano G (2017). Are all people with diabetes and cardiovascular risk factors or microvascular complications at very high risk? Findings from the risk and prevention study. Acta Diabetol.

[18] DeFronzo RA, Stonehouse AH, Han J, Wintle ME (2010). Relationship of baseline HbA1c and efficacy of current glucose-lowering therapies: a meta-analysis of randomized clinical trials. Diabet Med.

[19] Lapolla A, Frison V, Bettio M (2015). Correlation between baseline characteristics and clinical outcomes in a large population of diabetes patients treated with liraglutide in a real-world setting in Italy. Clin Ther.

[20] Toyoda M, Yokoyama H, Abe K, Nakamura S, Suzuki D (2014). Predictors of response to liraglutide in Japanese type 2 diabetes. Diabetes Res Clin Pract.

[21] Gautier JF, Martinez L, Penfornis A (2015). Effectiveness and persistence with liraglutide among patients with type 2 diabetes in routine clinical practice–EVIDENCE: a prospective, 2-year follow-up, observational, post-marketing study. Adv Ther.

[22] American Diabetes Association (2017). Standards of medical care in diabetes—2017. Diabetes Care.

[23] Inoue K, Maeda N, Fujishima Y (2014). Long-term impact of liraglutide, a glucagon-like peptide-1 (GLP-1) analogue, on body weight and glycemic control in Japanese type 2 diabetes: an observational study. Diabetol Metab Syndr.

[24] Kozawa J, Inoue K, Iwamoto R (2012). Liraglutide is effective in type 2 diabetic patients with sustained endogenous insulin-secreting capacity. J Diabetes Invest.

[25] Iwao T, Sakai K, Sata M (2013). Postprandial serum C-peptide is a useful parameter in the prediction of successful switching to liraglutide monotherapy from complex insulin therapy in Japanese patients with type 2 diabetes. J Diabetes Complicat.

